# Optimisation of Remote Monitoring Programmes in Heart Failure: Evaluation of Patient Drop-Out Behaviour and Healthcare Professionals’ Perspectives

**DOI:** 10.3390/healthcare12131271

**Published:** 2024-06-26

**Authors:** Maria Pagano, Francesco Corallo, Anna Anselmo, Fabio Mauro Giambò, Giuseppe Micali, Antonio Duca, Piercataldo D’Aleo, Alessia Bramanti, Marina Garofano, Placido Bramanti, Irene Cappadona

**Affiliations:** 1 IRCCS Centro Neurolesi Bonino-Pulejo, Via Palermo, S.S. 113, C.da Casazza, 98124 Messina, Italy; 2 Department of Medicine, Surgery and Dentistry, University of Salerno, 84081 Baronissi, Italy; 3 Faculty of Psychology, Università degli Studi eCampus, Via Isimbardi 10, 22060 Novedrate, Italy

**Keywords:** cardiac rehabilitation, remote monitoring, drop-out, cognitive behavioral analysis

## Abstract

Heart failure (HF) is a growing epidemic, affecting millions of people worldwide, and is a major cause of mortality, morbidity, and impaired quality of life. Traditional cardiac rehabilitation is a valuable approach to the physical and quality-of-life recovery of patients with cardiovascular disease. The innovative approach of remote monitoring through telemedicine offers a solution based on modern technologies, enabling continuous collection of health data outside the hospital environment. Remote monitoring devices present challenges that could adversely affect patient adherence, resulting in the risk of dropout. By applying a cognitive-behavioral model, we aim to identify the antecedents of dropout behavior among patients adhering to traditional cardiac rehabilitation programs and remote monitoring in order to improve the latter. Our study was conducted from October 2023 to January 2024. In the first stage, we used data from literature consultation. Subsequently, data were collected from the direct experience of 49 health workers related to both remote monitoring and traditional treatment, recruited from the authors’ workplace. Results indicate that patients with cardiovascular disease tend to abandon remote monitoring programs more frequently than traditional cardiac rehabilitation therapies. It is critical to design approaches that take these barriers into account to improve adherence and patient satisfaction. This analysis identified specific antecedents to address, helping to improve current monitoring models. This is crucial to promote care continuity and to achieve self-management by patients in the future.

## 1. Introduction

### 1.1. Heart Failure: A Growing Global Epidemic

Heart failure (HF) is a growing global epidemic affecting more than 37.7 million people worldwide [1] and it represents a major cause of mortality, morbidity, hospitalization, and poor quality of life. Heart failure is defined as a clinical syndrome characterized by the inability of the heart to pump blood through the body at a rate adequate for its needs, or to do so only at the cost of high filling pressures because of functional or organic abnormalities [2]. 

### 1.2. Promoting Recovery in Heart Failure Treatment: Key Role of Remote Monitoring

Cardiac rehabilitation (CR) is a key component of HF treatment as it aims to improve quality of life and reduce hospitalizations through education, exercise, stress management, and lifestyle modification [3]. Both in-person rehabilitation and remote monitoring aim to promote patient recovery [4]. Remote monitoring of heart failure patients through telemedicine is an approach that takes advantage of modern technologies to receive health care and continuously collect data on sick individuals outside the hospital setting [5]. This approach has led to a reduction in hospital admissions and length of stay because it allows for early intervention in case of changes in the patient’s health status [6]. In addition, it can be accompanied by psychological support services that help patients manage the stress and anxiety related to their medical condition [7]. In the elderly and their caregivers, remote monitoring increases peace of mind and reduces disease-related distress and anxiety related to physical distance from the hospital. Patients are actively involved through access to real-time data via apps or online platforms; this allows for more informed and responsible participation in their own well-being [8]. Four types of data can be monitored: (I) symptoms, e.g., shortness of breath and fatigue; (II) behaviors or events, e.g., medication compliance and physical activity; (III) biological data acquired noninvasively through external devices, e.g., body weight, blood pressure, heart rate, and ECG; (IV) biological data acquired invasively through implantable devices, e.g., intracardiac blood pressure [9]. The data provided by remote monitoring can promote patient empowerment by actively involving the patient in managing their health and making them more aware of the factors that influence their condition. 

### 1.3. Addressing Limitations and Preventing Abandonment of Remote Monitoring

Despite the progress and benefits of monitoring from the literature, limitations emerge that could be enhanced. Asynchronous contact, such as through email, could negatively affect the quality of patient-clinician interaction as decreased empathy and loss of meaningful nonverbal information. This can lead to feelings of loneliness, especially for those who find comfort and support in medical appointments. Remote monitoring devices may have a complex interface that is difficult for the elderly to understand, which could generate frustration and constant calls for help to caregivers. Patients with physical limitations may find it difficult to use remote monitoring dis-positive devices, especially if these are not designed with their specific needs in mind. For example, those with visual impairment may have difficulty viewing the screen and may require constant assistance from a caregiver. In addition, there may be problems with the Internet in rural areas, where the high-speed connection may be limited, subject to interruptions, or totally absent. These difficulties could prompt patients to drop out of the monitoring program. Finally, if patients do not perceive the importance of remote monitoring to their well-being or if they are not sufficiently motivated to take care of their health, they may be less inclined to continue participating.

Therefore, it is essential to design approaches that consider aspects that result in dropout behaviors to avoid discontinuation and improve patient satisfaction. The effectiveness of remote monitoring for heart failure patients could be increased through cognitive behavioral analysis. 

### 1.4. Cognitive Behavioral Analysis: Antecedents, Consequences and Function of Behavior

Cognitive behavioral theories tend to be linear, explaining why behavior may occur by considering a number of predictors and how these might influence the likelihood of a behavior [10]. One must look at what comes before, that is the antecedents (A), and what comes after, that is the consequence (C); such analysis can explain the function of the behavior (B). The analysis of antecedents allows one to identify the motivations that increase and decrease the occurrence of a behavior [11]. Therefore, if a certain behavior occurs frequently after a specific event, that event can be considered a significant antecedent. Similarly, observing the consequences of the behavior helps to understand how they influence its repetition: if a behavior produces a positive consequence, it is more likely to be repeated. This in-depth understanding of antecedent factors and consequences is essential for developing effective intervention strategies.

### 1.5. Aims

The objective of our study is to identify behavioral antecedents on which to base more effective models. Initially, this was conducted with a literature consult and successively by administering questionnaires to health care providers who participated in remote monitoring and traditional treatment.

## 2. Materials and Methods

**Figure 1:**
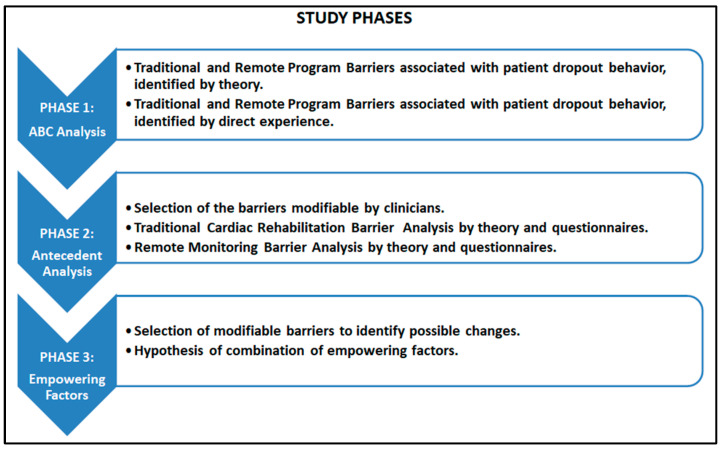
Study phases.

From October 2023 to January 2024, our research was conducted. In the present study, data from the literature and data from the direct experience of 49 health workers recruited at the authors’ workplace were used. See Figure 1 for a detailed description of the three phases of the study.

### 2.1. Phase 1: ABC Analysis

**Figure 2:**
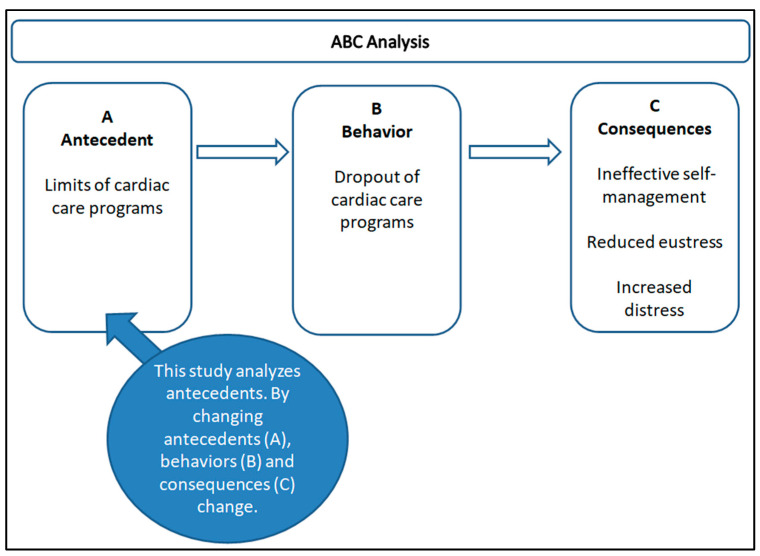
ABC Analysis.

We examined the scientific literature, consulting meta-analysis studies and systematic reviews in major databases such as PubMed and Cochrane, to analyze the participation of patients with heart failure and other cardiovascular diseases in rehabilitation and remote monitoring programs. Studies have identified possible causes of patients’ abandonment behavior. In addition, studies have assessed the factors that motivate patients to participate in traditional rehabilitation and remote monitoring. We collected professionals’ opinions through two specially prepared semi-structured online questionnaires ad hoc on the motivating and demotivating factors for patients’ participation in the relevant pathways expressed in the reviews analyzed [4,12]. One questionnaire was aimed at professionals with experience in traditional rehabilitation (doctors, nurses, and rehabilitators) and one questionnaire was aimed at professionals with experience in remote monitoring programs (doctors, nurses, rehabilitation, and telemedicine operators). Both questionnaires were completed anonymously and included a demographic section and a section of multiple-choice questions (ten multiple-choice questions for each questionnaire) with a Likert 0 to 5 scale on the strengths and weaknesses of the treatments analyzed. Finally, both questionnaires included two open-ended questions on the motivating and demotivating factors found in the patients. This analysis served to identify behavioral antecedents on which to build more effective models. The behavioral ABC concept analyzed is summarized in Figure 2.

### 2.2. Phase 2: Behavioral Antecedents

After the analyses in phase 1, we identified the behavioral antecedents of heart failure patients adhering to remote monitoring and conventional treatment. In particular, we selected modifiable barriers that can be manipulated by the clinicians.

### 2.3. Phase 3: Empowering Elements

Based on the previous steps, we hypothesized possible changes to be made and combined them with existing strengths to propose enhancing elements for monitoring programs.

## 3. Results

Below are the results of the literature consultation and those that emerged from the questionnaires on the direct experience of the 49 health workers involved.

### 3.1. Results of the Literature Consultation

Recent literature reviews and meta-analyses [4,12] show that the dropout rate for traditional cardiac rehabilitation ranges from 12% to 56%. In comparison, the dropout rate for remote monitoring shows even greater variability, ranging from 9% to 78% (see Table 1).

**Figure 3:**
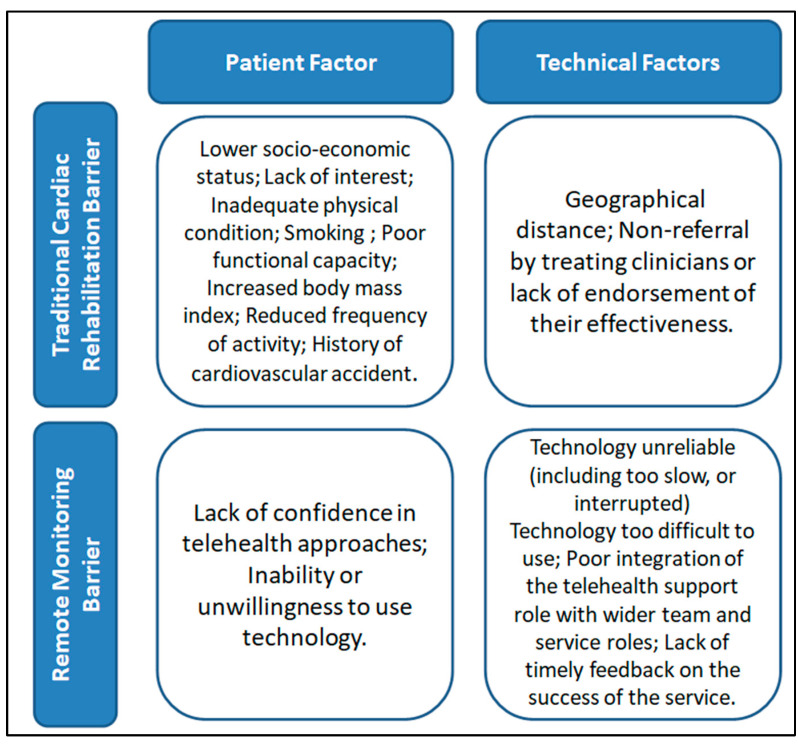
Results of the literature consultation. In traditional rehabilitation, patient-related barriers prevail. In remote monitoring, technology-related barriers prevail.

From the literature analysis, a higher dropout rate has emerged in remote monitoring programs compared to traditional rehabilitation. The reasons for dropout in both analyzed pathways were attributed to patient-related characteristics, as well as technical and logistical features (Figure 3). In addition, for traditional cardiac rehabilitation, patient-related causes were prevalent, while for remote monitoring programs, causes related to technical problems were prevalent.

### 3.2. Results from the Analysis of Professionals’ Direct Experience

We reviewed a total of 49 responses from health care providers: 28 with experience in traditional care and 21 with experience in remote monitoring. For a detailed description of socio-demographic characteristics, see Table 2.

### 3.3. Traditional Cardiac Rehabilitation

**Figure 4:**
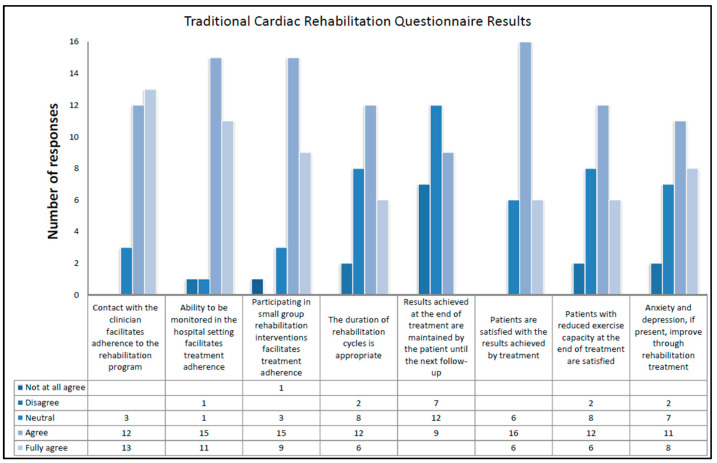
Results of questionnaires on traditional cardiac rehabilitation for each question.

The results of traditional cardiac rehabilitation align with findings from the literature. In particular, professionals believe that the facilitating elements include the following: contact with the clinician (89%), in-hospital monitoring (92%), rehabilitation in small groups (86%), and satisfaction with the outcomes achieved through traditional treatment (79%). Experts hold contrasting opinions on the facilitating role of the duration of rehabilitation cycles, the maintenance of results until the next follow-up, and patient satisfaction with reduced exercise capacity. Professionals are also divided on the effectiveness of traditional rehabilitation in improving anxiety and depression. Specifically, 7% strongly disagree on the improvement of anxiety and depression, 68% assert that physical rehabilitation has a positive impact, and the remaining 25% remain neutral on this matter. For a detailed description, see Figure 4

Each professional who participated in the study answered two open-ended questions about the motivating and demotivating aspects for the patient who wants to undertake traditional rehabilitation. For a detailed description of the responses, see Table 3.

Experienced professionals in the field say the most motivating aspects of continuing traditional cardiac rehabilitation treatments are related to physical recovery (46%), participation in small groups (25%), psychological support (18%), and direct contact with referring physicians (39%). In contrast, demotivating aspects relate to logistical difficulties such as distance from the rehabilitation site (36%), the presence of anxiety and depression (43%), and aspects related to the organization of the service (14%).

### 3.4. Remote Monitoring Programs

**Figure 5:**
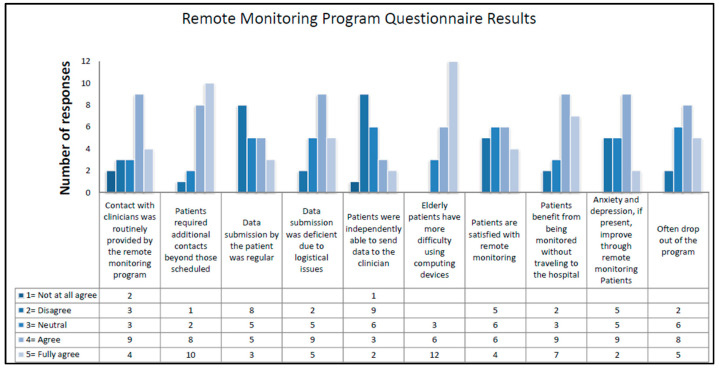
Results of questionnaires on remote monitoring programs for each question.

In this case, the results of remote monitoring programs align with those found in the literature analysis. It has been observed that remote monitoring programs involve routine contacts with clinicians (62%), although many professionals report that patients required additional contacts (86%). Overall, 66% of operators state that data submission was lacking due to logistical reasons, and patients were unable to independently send data collected by the device (48%), especially elderly patients (86%). A facilitating factor for participation in remote programs is the benefit of being monitored without having to visit the hospital. However, 61% affirm that dropout is frequent. Finally, 52% state that, if present, anxiety and depression improve with remote monitoring. Opinions on patient satisfaction with remote monitoring are favorable for 48%, neutral for 29%, and unfavorable for 23% (Figure 5).

Each professional who participated in the study answered two open-ended questions about the motivating and demotivating aspects of remote monitoring for the patient. For a detailed description of the responses, see Table 4.

Experienced professionals in the field say that the most motivating aspects of continuing remote monitoring programs are related to contact with physicians where provided (33%), reduction of anxiety due to continuous monitoring (29%), and reduction of geographic distances that promotes equity of access to care (14%). In contrast, demotivating aspects include the complexity of equipment and the process of sending data to the physician (57%), lack of contact with physicians (14%), and the presence of anxiety, depression, and stress (29%).

### 3.5. Proposal for Enhancement Elements

Based on the results obtained from remote monitoring and traditional treatment, we have identified factors on which the clinician can intervene through cognitive-behavioral analysis. The analysis is detailed in Table 5.

### 3.6. Identification and Implementation of Strategies for Improvement

**Figure 6:**
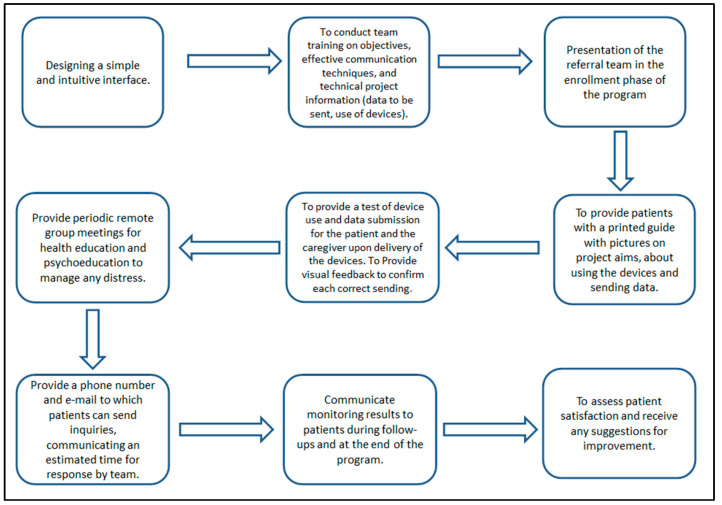
Elements of remote program enhancement.

This assessment allowed for the identification of reinforcing elements to address weaknesses and the inclusion of new elements deemed effective. For a detailed description of the reinforcing elements, see Figure 6.

## 4. Discussion

Our study analyzed the antecedents of the dropout behavior for two types of interventions. We examined the reasons for dropout in traditional cardiac rehabilitation programs to evaluate what motivates patients to complete the course. We also explored the reasons for dropout in remote monitoring programs to understand what leads patients to abandon the course. This examination allowed us to identify antecedents for intervention, enhancing current monitoring models. 

The results highlight that patients with heart failure and other cardiovascular conditions are more likely to abandon remote monitoring programs compared to traditional cardiac rehabilitation treatments [13,14]. 

### 4.1. Traditional Cardiology Rehabilitation and Remote Monitoring

Traditional cardiology rehabilitation is characterized by direct and regular contact between patients and clinicians, establishing a solid bond of trust. During these personal interactions, patients benefit from immediate emotional support, instant feedback on their condition, and comprehensive assessment of their physical condition [3,7]. This type of approach encourages active patient involvement in the treatment process and promotes greater adherence to the rehabilitation program [4].

Remote monitoring represents an innovative mode of care that allows the physician to monitor the patient’s health status remotely, exploiting the potential of telemedicine. This approach offers numerous advantages, such as the ability to collect real-time data and detect changes in the patient’s vital parameters early [5,7]. It also offers greater flexibility and convenience for the patient, reducing the need for frequent hospital or outpatient visits. This means reduced costs associated with travel to follow-up appointments [9]. The use of remote monitoring allows health services to expand the scope of cardiac rehabilitation services without increasing the workload of health care personnel or the use of resources [8]. This can help improve the accessibility and coverage of rehabilitation services, ensuring that more patients can benefit from quality care, regardless of their geographic location or willingness to travel to the hospital.

The integration of traditional approaches and remote monitoring could optimise patient outcomes by combining the personal relationship of traditional rehabilitation with the convenience of telemedicine. This balance allows for personalised attention during face-to-face interactions and constant remote monitoring. 

### 4.2. Determinants of Abandonment of Traditional Cardiac Rehabilitation and Remote Monitoring

Reasons for dropout in traditional cardiac rehabilitation are predominantly linked to patient-related factors [15] that clinicians cannot easily intervene in, such as age, socioeconomic status, and geographic distance, or can intervene minimally, such as reduced physical exercise capacity, resistance to lifestyle changes, and low interest [16,17,18]. An element that can be more readily addressed to reduce dropout in traditional cardiac rehabilitation is the establishment of a dedicated team [19], limiting excessive turnover of healthcare providers. Reasons for dropout in remote monitoring programs are mostly related to organizational and logistical factors that clinicians can modify, along with low patient motivation [4]. These factors include difficulties in technology use, especially among elderly patients, connectivity issues, and limited contact with designated clinicians [20,21]. Special attention should be given to scheduling contacts with clinicians. Even in Boriani et al.’s study [22], comparing exclusively outpatient monitoring with remote monitoring, the number of unscheduled visits for patients monitored remotely was four times higher than those in the standard monitoring group [23]. Participation in small groups can promote adherence to traditional cardiac rehabilitation treatments and can also be an effective coping strategy for patients with heart failure. The group allows patients to share their experiences and feelings with individuals facing similar challenges. This can reduce the sense of isolation and promote treatment adherence. Therefore, introducing periodic group meetings could influence the patient’s ability to engage in remote monitoring programs.

### 4.3. Application of Functional Behavioral Assessment to Reduce Patient Abandonment

Functional Behavioral Assessment (FBA) includes a descriptive process that identifies the role of antecedent variables before the behavior and the consequences that follow the analyzed problem behavior [24]. Improving the scheduling of monitoring programs reduces patient dropout. FBA techniques include various assessment tools, including interviews. A problem behavior can be improved by intervening in the antecedents or consequences that maintain it [10]. The cognitive behavioral approach is a methodology that combines the principles of cognitive and behavioral psychology to understand and modify dysfunctional behaviors. This approach has become an increasingly valuable tool used in a variety of clinical and organizational settings because behavioral analysis enables the planning of health services and interventions that improve the quality of care and effectiveness of treatment. In our study, it was found that, in traditional cardiac rehabilitation, the factors that can be changed solely by the physician are: lack of referral from treating physicians, lack of approval of physician effectiveness, and lack of interest. These factors can be modified through: training on effective communication with the patient, reduction of physician turnover, and implementation of health education and psychoeducation pathways. In remote monitoring, factors that can be modified solely by the physician include: lack of confidence in telemedicine approaches, preference for direct contact with the physician, inability or reluctance to use technology, unreliable technology, technology too difficult to use, and poor integration of the telemedicine support role with the broader team and service roles. These elements can be changed through: training on the methods and purposes of the programs prior to recruitment, implementing synchronous contact with a team of referring physicians, developing simple and intuitive usability, verifying the reliability of the network through periodic testing, and introducing immediate visual feedback to confirm correct data submission.

### 4.4. Limitations and Strengths

To our knowledge, our study is the first to propose enhancement elements for remote monitoring programs based on the limitations identified in the literature and the direct experience of healthcare professionals.

The main limitation of our study is not collecting the direct perspective of patients, which would undoubtedly be valuable to identify key issues. Potential errors and variability in health care providers’ responses are also a limitation. Providers may not be fully aware of all the difficulties faced by patients or may misinterpret the causes of their dropout. Through analysis of patients’ experiences and perceptions, it would be possible to identify specific factors contributing to the difficulty of adhering to monitoring programs. These factors could include technical problems, lack of adequate support, difficulty understanding instructions, or lack of motivation. Understanding the personal challenges and motivations underlying dropout behavior would provide important information for improving patient compliance and the effectiveness of remote monitoring programs in cardiac rehabilitation. Another limitation is the absence of the perspective of caregivers. These people, often family members or close acquaintances of patients, play a crucial role in the process of care and assistance. Their perspective provides a more comprehensive view of the challenges faced by patients, whether in traditional cardiac rehabilitation or remote monitoring. They accompany patients in their daily experiences, supporting them with difficulties such as technical problems and closely observing their progress and needs that are not always expressed directly.

Additionally, an analysis comparing traditional cardiac rehabilitation and remote monitoring has the inherent limitation of comparing different types of treatments, although physical activity is often monitored remotely as well [25,26]. These two approaches are the most widely used to support patients with heart failure and other cardiovascular conditions in recovering daily life activities [4]. In both remote monitoring and traditional treatment of cardiovascular disease, the goal is to provide patients with the tools and support they need to effectively manage their condition. In this way, it is possible to reduce distress (negative stress) [27] and increase eustress (positive stress) in patients. In remote monitoring, patients can experience a feeling of safety and well-being through regular monitoring of their vital parameters. In traditional treatment, patients are engaged in structured physical exercises and group therapy sessions that can help improve mood and reduce stress. In this way, reduced discomfort and increased eustress promote the patient’s overall well-being [28]. Cardiovascular diseases are a major social and economic problem [29] and remain the diseases with the highest mortality and morbidity rates [30].

### 4.5. Future Perspectives

Future perspectives on heart failure and other cardiovascular conditions management are moving towards patient self-management [21]. Self-management would allow patients to be independent in monitoring symptoms, understanding the need for medication readjustment, and discerning when to consult a physician [31]. However, achieving the ultimate goal of self-management requires guiding the patient to understand their clinical condition and the changes that occur over time. Following patients with standardized therapeutic and care pathways using traditional, remote, or integrated approaches can facilitate the achievement of self-management.

## 5. Conclusions

Traditional cardiac rehabilitation represents a historically valid approach in the recovery of physical capabilities and quality of life for patients with heart failure and other cardiovascular conditions. This treatment has the advantage of direct contact with clinicians and is well-tolerated by patients, with a lower treatment dropout rate compared to remote monitoring. Remote monitoring programs have developed more recently and offer the advantage of monitoring patients while overcoming logistical barriers such as geographic limitations. However, remote monitoring programs experience a higher dropout rate due to technical difficulties in using the equipment and the absence or intermittence of contact with clinicians. Implementing remote programs and preventing patient dropout is crucial because they serve as a valuable guide to steer patients towards self-management of the disease.

## Figures and Tables

**Table 1:** Literature consultation on dropout rates on traditional cardiology rehabilitation and remote monitoring.

References Review Present in the Dairy	Type of Rehabilitation	Number of Studies Analyzed	Range Years	Minimum Dropout Rate	Maximum Dropout Rate
Turk-Adawi et al. [12]	Traditional cardiology rehabilitation	8	1989 to 2011	12%	56%
Hannan et al. [4]	Remote monitoring	9	2009 to 2018	9%	78%

**Table 2:** Socio demographic characteristics.

Socio-Demographic Variables	Traditional Cardiac Rehabilitation	Remote Monitoring Program
Male	13	8
Female	15	13
Age ≤ 30 years	2	2
Age 31–50 years	23	16
Age ≥ 50 years	3	3
Work seniority 0–10 years	10	5
Work seniority 11–20 years	13	13
Work seniority 21–30 years	4	3
Work seniority > 30 years	1	/
Bachelor’s Degree	8	3
Master’s Degree	13	13
Postgraduate Degree	7	5

**Table 3:** Responses to open-ended questions on the motivating and demotivating aspects of traditional rehabilitation.

Indicate from Your Experience the Aspect That Most Motivates the Patient to Treatment.	Indicate from Your Experience the Aspect That Does Not Motivate the Patient to Treatment.
1. Physical and mental recovery	1. Logistical difficulties
2. Participation in groups	2. Depression
3. Functional recovery, empathy of carers, the expectation of a better quality of life.	3. Initial mistrust, socio-cultural barriers, resistance to lifestyle change, logistical difficulties in reaching the venue.
4. Physical recovery, contact with the clinician, participation in groups	4. Logistical difficulties, anxiety, depression
5. Hospitality and professionalism	5. Lack of information on the rehabilitation treatment to be carried out
6. Physical recovery, contact with the clinician, participation in groups	6. Logistical difficulties, anxiety, stress, depression
7. Physical recovery, mood	7. Anxiety, motivation
8. Participation in groups	8. Anxiety
9. Direct contact with clinical staff	9. Difficulties in reaching the facility, especially if physically distant
10. Contact with the clinician	10. Logistical difficulties
11. Direct contact with the reference team	11. Logistical difficulties in reaching the venue
12. Physical recovery and psychological support	12. Discouragement and lack of organization in rehabilitation activities
13. Participation in small groups increases motivation and adherence	13. Improving the organization
14. /	14. /
15. Physical recovery	15. Logistical difficulties
16. Contact with the clinician	16. Logistical difficulties
17. Physical recovery	17. Stress
18. Contact with the clinician	18. Stress
19. Recovery of physical performance, rehabilitation activity in groups	19. Still being busy with work and not reconciling with rehabilitation schedules.
20. Contact with the reference team, participation in groups, physical recovery as far as possible	20. Remoteness and difficulty in reaching the venue
21. Therapeutic interaction, positive psychological attitude	21. Depression
22. Contact with the clinician	22. Depression
23. Physical recovery	23. Depression
24. Contact with the clinician	24. Logistical difficulties
25. Contact with the clinician	25. Depression
26. The pre-injury lifestyle	26. Review
27. Physical recovery	27. Commitment required
28. The perception of being taken care of and being looked after	28. Lack of confidence in clinical treatment

**Table 4:** Responses to open-ended questions on the motivating and demotivating aspects of remote monitoring programs.

Indicate from Your Experience the Aspect That Most Motivates the Patient to Remote Monitoring.	Indicate from Your Experience the Aspect That Does Not Motivate the Patient to Remote Monitoring.
1. Having the peace of mind that it is monitored by a professional	1. The complexity of the equipment
2. Contact with the clinician	2. Difficulties in handling equipment
3. None	3. Technical and logistical difficulties
4. Difficulties in moving, potential convenience of the instrument, contact with the referring clinician.	4. Excessively high number of instruments, network issues, intermittent contact with the clinician.
5. Contact with the clinician	5. Anxiety, stress, depression
6. Contact with the clinician	6. Anxiety, depression
7. Contact with the clinician	7. Anxiety
8. The especially older patient prefers contact with the clinician	8. Some patients cannot handle devices
9. The possibility of being followed by clinicians without the need to travel to the facility	9. The ‘duty’ to send parameters at set times, or at least punctual slots
10. Contact with the clinician	10. Stress
11. Monitoring makes the patient feel calmer because they know they are constantly being monitored. 24-h monitoring also makes family members feel more relaxed.	11. Complex applications that make it difficult to use, especially for elderly patients. Connection problems.
12. Not feeling alone	12. The thought of being abandoned by the clinician
13. Ease with which the clinician can reach the patient	13. Facial contact cannot be replaced with remote
14. /	14. /
15. Contact with the clinician	15. Stress
16. The reduction of anxiety because they are constantly monitored	16. Difficulties in using platforms
17. Functional alternative	17. Stress
18. Contact with the clinician	18. The obligation to send
19. Knowing that there is continuous monitoring of their health status	19. Equipment too complicated to send data
20. Logistical difficulties, contact with referring clinicians if foreseen	20. Connection difficulties, asynchronous and untimely communication
21. Equity of access	21. Technological difficulties

**Table 5:** Behavioral antecedents and proposed improvement.

Type of Intervention	Antecedent	Modifiable Exclusively by Clinicians	How to Change
Traditional Cardiac Rehabilitation	Non-referral by treating clinicians	Yes	Training in effective patient communication; Avoiding clinician turnover.
	Lack of endorsement of clinicians effectiveness	Yes	Training in effective patient communication
	Lower socio-economic status	No	/
	Lack of interest	Yes	Implement health education and psychoeducation pathways
	Inadequate physical condition	No	/
	Geographical distance	No	/
	Smoking	No	/
	Poor functional capacity	No	/
	Increased body mass index	No	/
	History of cardiovascular accident	No	/
	Reduced frequency of activity	No	/
			
Remote Heart Failure Monitoring	Lack of confidence in telehealth approaches	Yes	Training on methods and purpose of programs before recruitment
	Preference for direct contact with clinician	Yes	Implement synchronous contacts with a team of referring clinicians
	Inability or unwillingness to use technology	Yes	Define simple and intuitive usability
	Technology unreliable (including too slow, or interrupted)	Yes	Verify network reliability through periodic testing
	Technology too difficult to use (user interface design, accessibility features, complexity of procedures, integration with other devices)	Yes	Define simple and intuitive usability
	Poor integration of the telehealth support role with wider team and service roles	Yes	Implement synchronous contacts with a team of referring clinicians
	Lack of timely feedback on the success of the service	Yes	Introduce immediate visual feedback to confirm correct data submission

## Data Availability

Datasets are available to download on request. Requests should be directed to the corresponding author: Francesco Corallo, francesco.corallo@irccsme.it.
